# Supplementary material for: Seminoma and Embryonal Carcinoma Footprints Identified by Analysis of Integrated Genome-Wide Epigenetic and Expression Profiles of Germ Cell Cancer Cell Lines
Source: PLoS One. 2014 Jun 2;9(6):e98330. doi: 10.1371/journal.pone.0098330 (PMC4041891; doi:10.1371/journal.pone.0098330)
Supplement: File S1 — ZIP file containing DMRforPairs output for significant regions. Please start from the html files. (ZIP) [file pone.0098330.s008.zip › figures/33084.pdf]

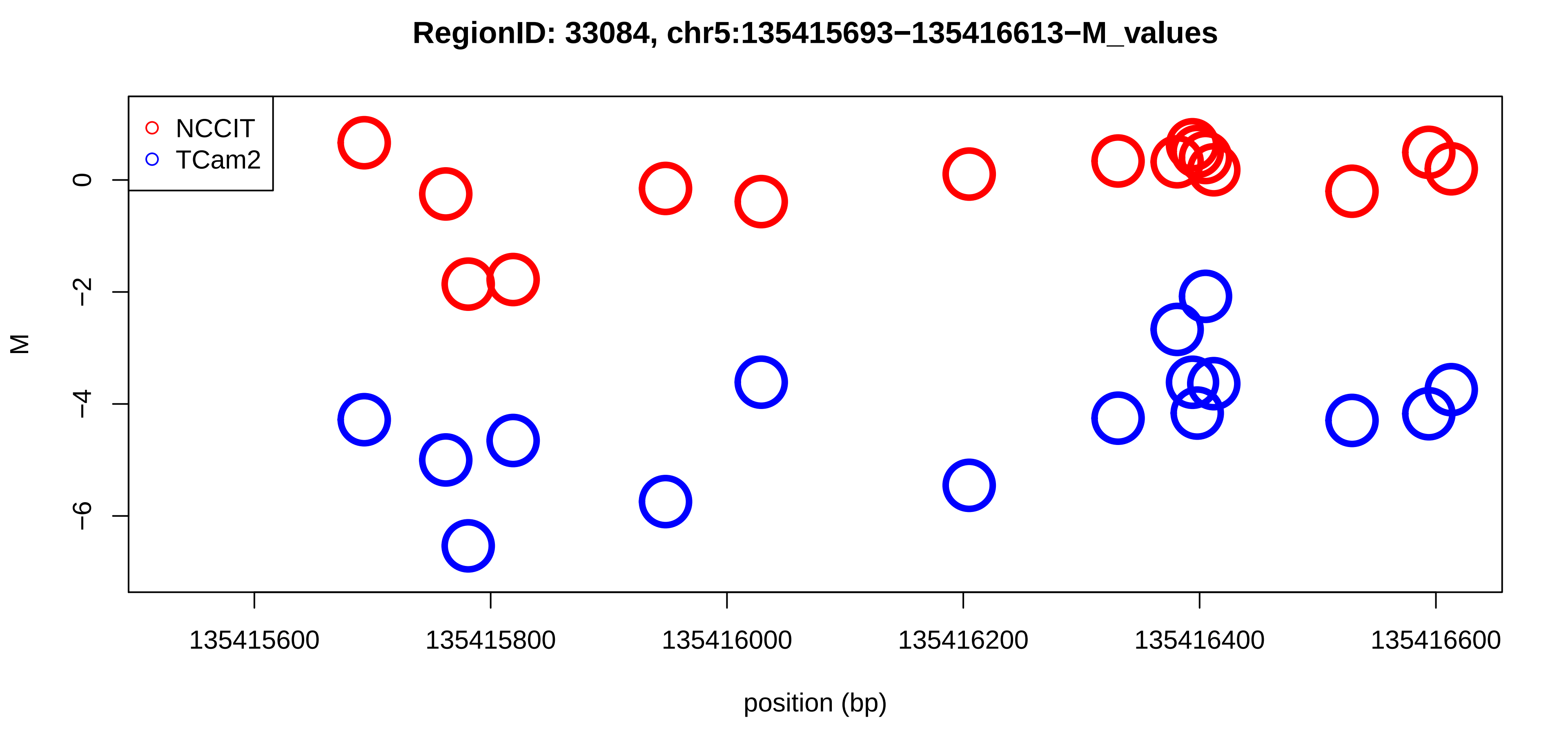
RegionID: 33084, chr5:135415693−135416613−M_values
NCCIT
TCam2
M
0
−2
−4
−6
135415600
135415800
135416000
135416200
135416400
135416600
position (bp)

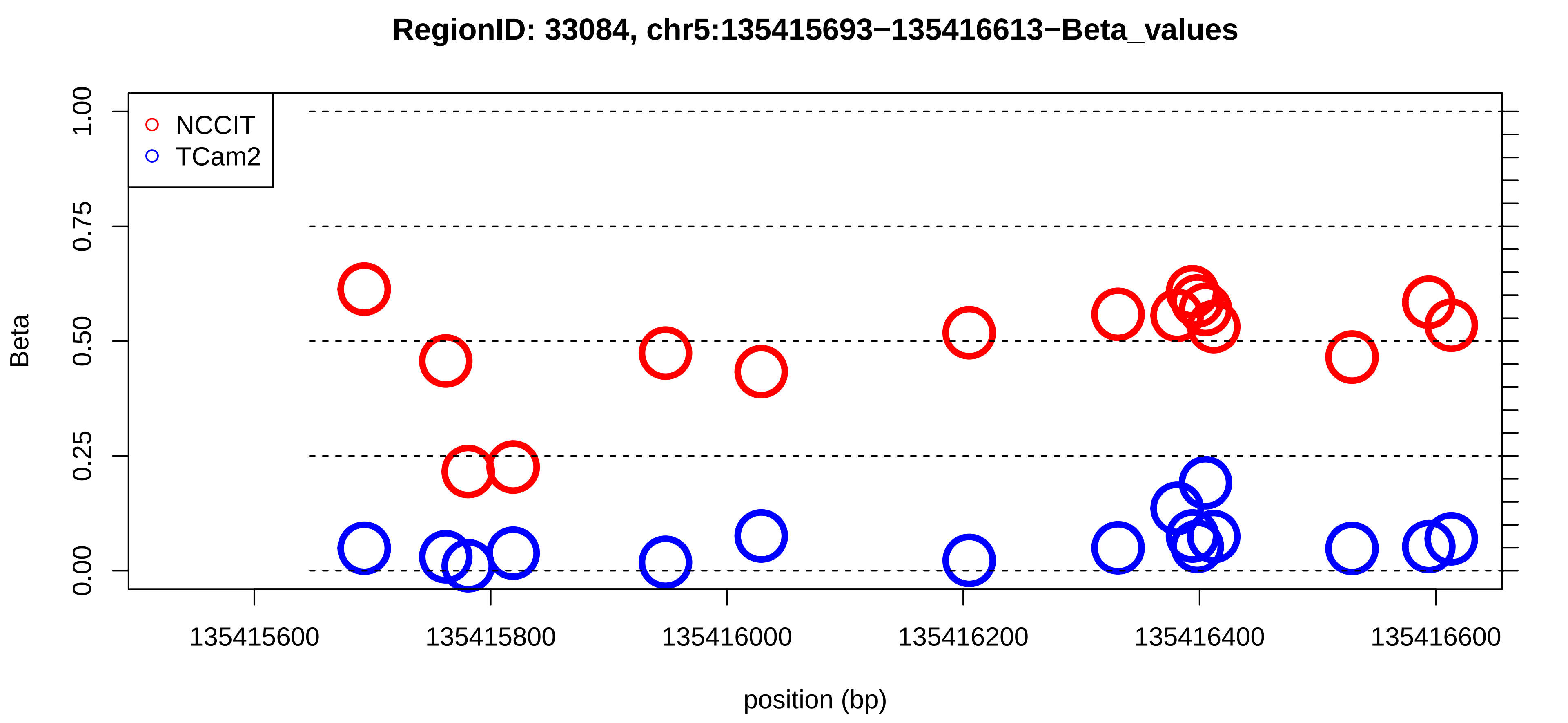
RegionID: 33084, chr5:135415693−135416613−Beta_values
NCCIT
TCam2
Beta
0.00
0.25
0.50
0.75
1.00
135415600
135415800
135416000
135416200
135416400
135416600
position (bp)

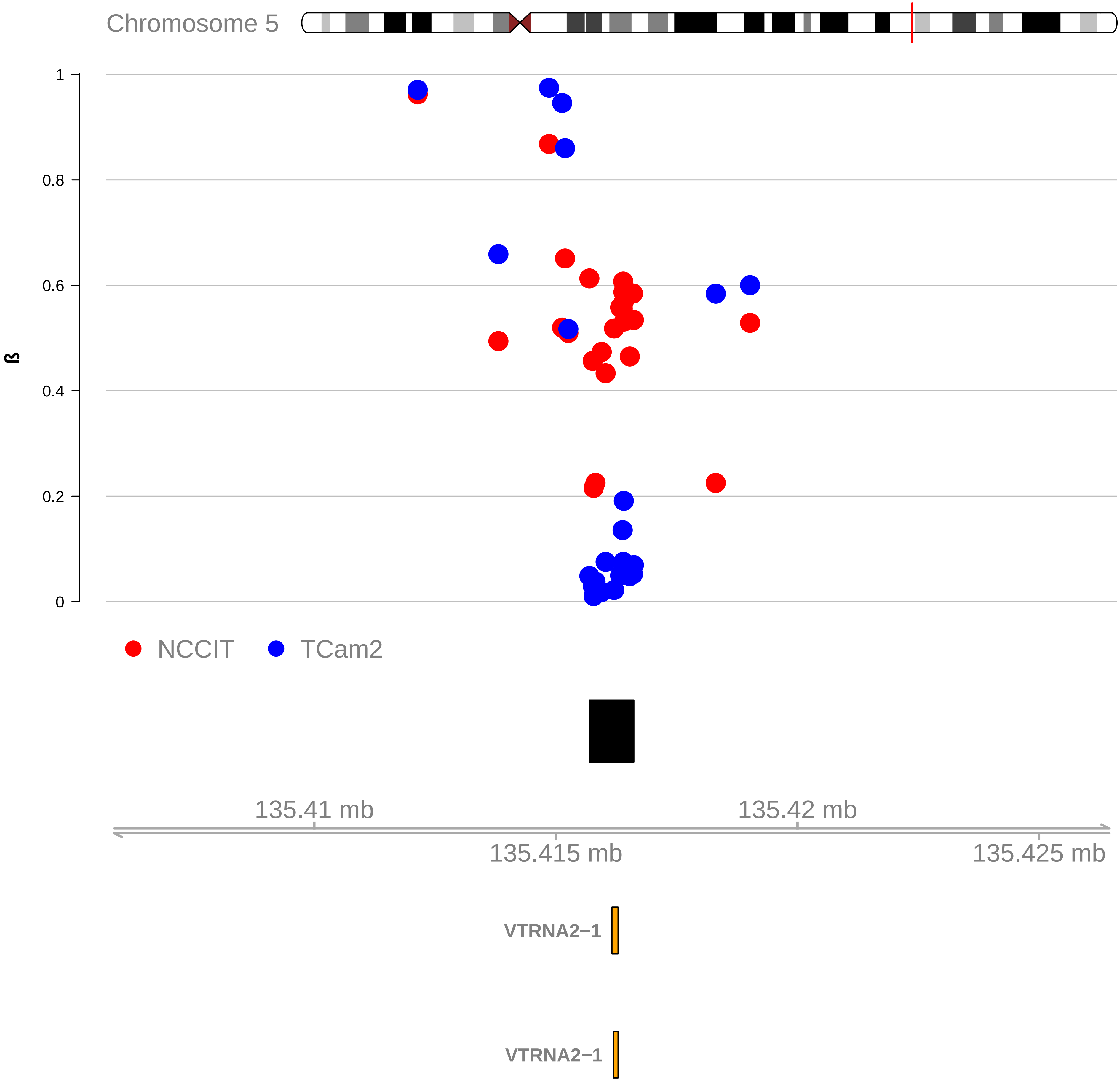
Chromosome 5
1
0.8
0.6
0.4
0.2
0
ß
NCCIT
TCam2
135.41 mb
135.42 mb
135.415 mb
135.425 mb
VTRNA2−1
VTRNA2−1
